# Age-dependent gene expression of *Calliphora vicina* pupae (Diptera: Calliphoridae) at constant and fluctuating temperatures

**DOI:** 10.1007/s00414-021-02704-x

**Published:** 2021-09-27

**Authors:** K. Hartmann, E. Herrmann, J. Amendt, M. A. Verhoff, R. Zehner

**Affiliations:** 1 Institute of Legal Medicine, University Hospital Frankfurt, Goethe University, Frankfurt am Main, Germany; 2 Department of Aquatic Ecotoxicology, FB Biowissenschaften, Goethe University, Frankfurt am Main, Germany; 3 Institute of Biostatistics and Mathematical Modeling, Goethe University, Frankfurt am Main, Germany

**Keywords:** Forensic entomology, Blow flies, Age estimation, Analysis tool, Post-mortem interval

## Abstract

Estimating the age of the developmental stages of the blow fly *Calliphora vicina* (Diptera: Calliphoridae) is of forensic relevance for the determination of the minimum post-mortem interval (PMI_min_). Fly eggs and larvae can be aged using anatomical and morphological characters and their modification during development. However, such methods can only hardly be applied for aging fly pupae. Previous study described age estimation of *C. vicina* pupae using gene expression, but just when reared at constant temperatures, but fluctuating temperatures represent a more realistic scenario at a crime scene. Therefore, age-dependent gene expression of *C. vicina* pupae were compared at 3 fluctuating and 3 constant temperatures, the latter representing the mean values of the fluctuating profiles. The chosen marker genes showed uniform expression patterns during metamorphosis of *C. vicina* pupae bred at different temperature conditions (constant or fluctuating) but the same mean temperature (e.g. constant 10 °C vs. fluctuating 5–15 °C). We present an R-based statistical tool, which enables estimation of the age of the examined pupa based on the analysed gene expression data.

**Supplementary Information:**

The online version contains supplementary material available at 10.1007/s00414-021-02704-x.

## Introduction

The most important application of forensic entomology is the estimation of the minimum post-mortem interval (PMI_min_). This is based on the age estimation of the juvenile stages of necrophagous insects, which usually colonise the body very soon after death. Blow flies have special significance here, as they are often the first colonisers of a corpse. Their age therefore corresponds most closely with the actual time of death and provides the most valuable PMI_min_ [1, 2]. They pass through four developmental stages: egg, larva, pupa and imago [1]. There are established methods to determine the age of blow fly eggs and larvae based on anatomical and morphological changes. In the case of eggs, these changes can be visualised using histological analysis or light microscopy [3, 4]. To estimate the age of larvae, measurement of the age-dependent length or weight and comparison to species-specific reference data is used [5–7]. However, upon completion of feeding, the post-feeding larvae migrate from the corpse to pupariate [8], and therefore, due to the variable decrease of size during this stage, length measurement cannot be used for age estimation of those larvae without considering behavioural features (such as moving away from and not staying on the body).

After pupation, morphological methods could be used for age estimation if clear landmarks are defined. But such landmarks are not easy to define and inhere a high degree of subjectivity [9, 10]. To circumvent this, several molecular age markers for pupae of flesh flies [11, 12] and blow flies [13–17] have been described. Molecular analysis provides quantitative and accurate gene expression data and can be standardised. For this, the expression levels of various genes were characterised during pupal development. The measurement of the activity of these age-related genes sheds light on the age of the pupa. Differential expressed genes are promising as a potential age indicator during metamorphosis of flies. The quantitative analysis of these transcripts shows up- and downregulation of the genes. But due to the often low quantitative differences in expression within one gene (fold change values < 15 between different age landmarks) and the high interindividual variation, the generation of reliable age determination data is not easy.

Recently, quite larger different gene expression levels of *C.* *vicina* were detected during metamorphosis by means of a de novo transcriptome analysis [18, 19]. Markers for 15 different developmental stages of *C. vicina* pupae were characterized, which corresponds to each day during pupal development at 17 °C. These markers exhibit fold change values between approximately 10 and 40,000 compared to the conditions in the very early pupa, which is a much higher change in gene expression than observed for other age-dependent transcripts. Consequential qPCR assays for each of these markers, called A1/ A2–O1/ O2, have been validated for three different constant breeding temperatures within a range usually found in relevant forensic scenarios: 17 °C, 20 °C and 25 °C. The relative expression patterns of every marker during metamorphosis—except one—are similar for each temperature and corresponds to the transcriptome data, confirming that the selected age markers are independent of the examined constant temperatures [18, 19].

However, no studies have been conducted so far to examine those markers at fluctuating temperatures, although this represents a more realistic condition, particularly in outdoor scenarios. This is an important aspect, because it is not known whether the gene expression levels of the age markers are the same under constant and fluctuating temperature conditions. Various development studies proofed an effect of fluctuating versus constant temperature conditions on the development time of different insects [20–26]. Thus, accelerated development under fluctuating temperatures was observed for different blow flies such as *Calliphora vomitoria*, *Protophormia terraenovae* and *Lucilia sericata* [20, 26], whereas diminished development was observed in *Aldrichina grahami* and *C. vicina* compared to constant temperatures, respectively [23, 25, 26].

When determining their development or age, it should be noted that the development time of blow flies is depending on species-specific influence of the temperature. Greenberg and Kunich [27] assumed that the relation between rate of development and temperature is linear within a certain temperature range, in which development is possible. Because of this, the physiological age is given in accumulated degree hours (ADH) or accumulated degree days (ADD) to compare the development at different temperature conditions. ADH or ADD represents the summation of a specific amount of heat for development of an insect above its species-specific lower development threshold. There are hints that the amount of necessary ADD for the complete development may be temperature dependent [8]. Thus, it has already been described that the ADD required for development are lower than calculated near the lower temperature threshold and higher than calculated at or above the temperature optimum [24, 28].

Therefore, in the current study, the development of *C. vicina* bred under different fluctuating and constant temperatures above the lower development threshold to the upper development threshold was examined. Three temperature ranges were analysed, representing the lower, middle and upper temperature development range of *C. vicina*. The compared breeding conditions applied in the present study were constant temperatures of 10 °C, 20 °C and 30 °C, as well as uniformly fluctuating temperatures with corresponding mean values, i.e. 5–15 °C, 15–25 °C and 25–35 °C. Moreover, it was investigated whether the gene expression profiles of the pupae is just depending on the physiological age (ADD ≙ %-development at given temperature), regardless if environmental temperature is constant or fluctuating during development.

In addition, an R-based statistical tool was established, which enables age estimation based on the comparison of a certain gene expression pattern with the pattern of all age markers at all tested development stages. Therefore, an essential reference database with the gene expression data of the pupae bred at the mentioned constant and fluctuating temperature conditions was created as a basis for the age prediction tool. For validation of the age prediction tool, the gene expression data of *C. vicina* pupae of an outdoor breeding were exploited.

## Materials and methods

### Breeding and sampling

*C. vicina* of established stocks at the Institute of Legal Medicine in Frankfurt am Main, Germany, were used for this study. For oviposition, a piece of pig liver was put into the cage for 3 h. Thereafter the eggs were incubated at 25 °C ± 1 °C for 24 h. After eclosion, batches of 40 larvae were transferred to 40 g minced meat (50% pork/50% beef) and placed in a 200-ml plastic box. Depending on the experimental design (different temperature conditions lead to different sampling times and therefore different numbers of larvae per experiment), a total of approximately 300 to 600 larvae were used. The larvae were bred without light at constant temperatures of 10 °C (CV10) and 30 °C (CV30) as well as fluctuating temperatures 5–15 °C (CV5–15), 15–25 °C (CV15–25) and 25–35 °C (CV25–35), respectively; temperature tolerance was ± 1 °C. The fluctuating temperatures were such that their profiles resulted in the same ADD as for the constant temperatures. In addition to our own measurements, data collected at a constant temperature of 20 °C (CV20) by Zajac et al. [19] were used as a comparative data for CV15–25. Temperature modulation within 24 h initially started at the lower temperature and was maintained for 9 h, after which the temperature was increased to the higher temperature within 3 h. After a further 9 h, the temperature was again decreased to the lower temperature within 3 h, completing 24 h. As soon as the first pupae were found, they were sorted out until the day when most of the larvae had pupated. This day was the beginning of collection: five pupae of every breeding were sampled every 24 h (48 h for the outdoor breeding (CVO)), homogenised in TRIzol (TRI Reagent®, Sigma-Aldrich, Merck KGaA, Darmstadt, Germany) and stored until further processing at − 20 °C. Sampling was terminated with the eclosion of the first adult fly in the respective breeding series. Due to differences in the time required for total development at the different temperatures, a different number of pupae have been analysed for each temperature condition: CV10, *n* = 70; CV5–15, *n* = 30; CV20, *n* = 60; and CV15–25, *n* = 70. CV25–35 and CV30 were excluded from the further analyses due to mortality. In addition, an outdoor breeding (CVO) was carried out to validate the age prediction tool. Here the temperature range was between 8 and 27 °C with a mean of 15 °C (Supplementary Information, Table S1).

### RNA isolation and RNA quantification

After homogenisation of the pupa in 500 µl TRIzol, total RNA was extracted according to the TRI Reagent® Protocol (Sigma-Aldrich, Merck KGaA, Darmstadt, Germany). The precipitated RNA was dried for 5–10 min at 50 °C. Afterwards the RNA pellet was dissolved in RNA Storage Solution (Thermo Fisher Scientific, Waltham, USA) and stored at − 20 °C. Digestion of possible co-extracted DNA was prophylactically performed according to the TURBO DNA-*free*™ Kit manufacturer’s instructions (Invitrogen™, Thermo Fisher Scientific). Total RNA was quantified with the NanoDrop™ 1000 Spectrophotometer (Thermo Fisher Scientific).

Possible genomic DNA contaminations were detected by a highly sensitive amplification of the cytochrome c oxidase subunit I gene with universal primer [29]. PCR was performed using 1 µl extract and the AmpliTaq™ DNA Polymerase with Buffer I (Applied Biosystems™, Thermo Fisher Scientific) according to the manufacturer’s protocol with 1 U of polymerase in a total reaction volume of 25 µl. The thermal cycling conditions were 94 °C for 1 min, 5 cycles of 94 °C for 1 min, 45 °C for 1.5 min, 72 °C for 1.5 min, followed by 35 cycles of 94 °C for 1 min, 50 °C for 1.5 min, 72 °C for 1 min and a final extension at 72 °C for 8 min [18]. By 2.5% agarose gel electrophoresis, it was audited if PCR products occurred as hints for contaminating genomic DNA. In case of detected COI amplification, a DNA digestion was performed again.

### qPCR-based gene expression profiling

The gene expression levels of *C. vicina* pupae were analysed by amplification of molecular age markers, two for each of 15 developmental stages (labelled A1/A2–O1/O2) originally described in Zajac et al. [19] with the exception of markers E1 and E2, due to the small quantitative differences in expression (fold change values < 10). For markers D1 and D2, new primers were designed and established in comparison to Zajac et al. [19] for better performance (Supplementary Information, Table S2). The target sequences of the markers and the reference gene were annotated using blastx (Basic Local Alignment Search Tool). Almost all markers show homologies to the blow fly *Lucilia cuprina*, the closest relative with published transcriptome, but their functions are not yet known in detail. (Supplementary Information, Table S3). The validation of the molecular age markers was carried out by one-step RT-qPCR. The RT-qPCR was performed using the EXPRESS One-Step SYBR™ GreenER™ Kit, universal (Invitrogen™, Thermo Fisher Scientific) according to the manufacturer’s protocol in a reduced reaction volume of 10 µl, containing 40 ng total RNA, 100 nM ROX Reference Dye and 200 nM of each primer. For amplification, the standard cycling programme of the kit was performed on a 7500 Real-Time PCR System (Applied Biosystems™, Thermo Fisher Scientific).

### Data analysis

For the analysis of RT-qPCR, the 7500 Software v2.3 (Applied Biosystems™, Thermo Fisher Scientific) was used. Gene expression was normalised to the reference gene R2, which shows a constant expression throughout the metamorphosis [19]. The relative quantification of the gene expression of the markers was calculated using the comparative Ct method (ΔΔ Ct method) using DataAssist Software v3.01 (Applied Biosystems™, Thermo Fisher Scientific). The average gene expression of the 5 pupae on day 1 of pupation was used as the reference value.

The graphical visualisation of the analysed data was performed using GraphPad Prism 8.2.1 (GraphPad Software, San Diego, USA). The gene expression data of the age markers of the reference samples bred under the defined conditions represent the reference data sets for the age prediction tool.

### Age prediction tool

For age prediction of a pupa on the basis of the determined gene expression data, a tool was generated by means of the statistical programming language R [30] using RStudio Version 1.1.456 [31]. This tool consists of two scripts (available on request). The first script contains the gene expression data collected in the present study and acts as a reference database. For this, the gene expression data of each breeding were analysed separately in a first strategy. The mean values for each pupae age were calculated, and the respective cubic spline interpolation of each marker was computed. Thereby every marker received its own specific function. The overall fit over all samples was then calculated with a leave-one-out method. Subsequently, the 50%, 75% and 95% confidence interval (CI) were calculated. Beneath the input of the data of the individual breeding also calculations of combined data sets were tested: CVcold (includes CV10 and CV5–15), CVwarm (includes CV20 and CV15–25) and CVall (includes all four breeding).

The second script was then used for estimating an age interval of a pupa of unknown age. For this, its estimated gene expression data (fold change values against pupal development day 1) were be imported into the tool and compared to the analysed reference data of script 1. In this process, the distance function of the sample in question was calculated. The resulting confidence intervals cover the true age with a corresponding probability of 50%, 75% or 95%.

## Results

### Development of *C. vicina*

Successful breeding was observed at a constant temperature of 10 °C (CV10) and at fluctuating temperatures of 5–15 °C (CV5–15) and 15–25 °C (CV15–25). When breeding at fluctuating temperatures of 25–35 °C (CV25–35), the larvae died during larval development. At a constant temperature of 30 °C (CV30), all larvae pupated, but no imagos hatched. Therefore, CV25–35 and CV30 were excluded from the further analyses. Consequently, gene expression levels of 225 *C. vicina* pupae of the successful breeding have been analysed: CV10, *n* = 70; CV5–15, *n* = 30; CV20, *n* = 60; and CV15–25, *n* = 70.

The percentage time for development of the cold breeding (CV10 and CV5–15) was 41% and 42% for larval development and 58% and 59% for pupal development. For CV20 the percentage time for development from oviposition to pupation was 43% and from pupation to hatch 57%, whereas 35% for larval and 65% for pupal development were observed for CV15–25 (Table 1). The developmental period of insects correlates with specific environmental temperatures as expected. Therefore, the physiological age is indicated in ADD, which is the product of the summation of temperature (in °C) above the lower development threshold and time (in days) [8]. Every species has an individual thermal summation value necessary for total development. The required ADD of *C. vicina* for the complete development, from oviposition to eclosion of imago, is 388, using a lower development threshold of 2 °C [32]. However, the different breeding of *C. vicina* had different thermal summations. The colder the temperature, the higher the necessary thermal summation: Breeding CV10 took 69 days from oviposition to hatching of imago, which corresponds to 567 ADD (= (10 – 2) °C * 69 days). CV5–15 took 59 days for the complete development; i.e. 487 ADD. The thermal summation for CV15–25 was 365 ADD at 20 days, and for CV20, it was approximately 379 ADD at 21 days (Table 1).

**Table 1:** Developmental times and required ADD of each breeding of *C.* *vicina*. ADD was calculated using a lower development threshold of 2 °C [32]

Breeding	Experimental conditions (°C)	Days from oviposition to pupation (required ADD)	Days from pupation to hatch (required ADD)	Days from oviposition to hatch (required ADD)
CV10	10	**28** (41%) (239)	**41** (59%) (328)	**69** (100%) (567)
CV5–15	5–15	**25** (42%) (215)	**34** (58%) (272)	**59** (100%) (487)
CV20^a^	20	$\overline{x }$= **9** (43%) ($\overline{x }$ = 169)	$\overline{x }$= **12** (57%) ($\overline{x }$ = 210)	$\overline{x }$= **21** (100%) ($\overline{x }$ = 379)
CV15–25	15–25	**7** (35%) (131)	**13** (65%) (234)	**20** (100%) (365)

^a^Data from[19]

### Gene expression profiling

**Fig. 1:**
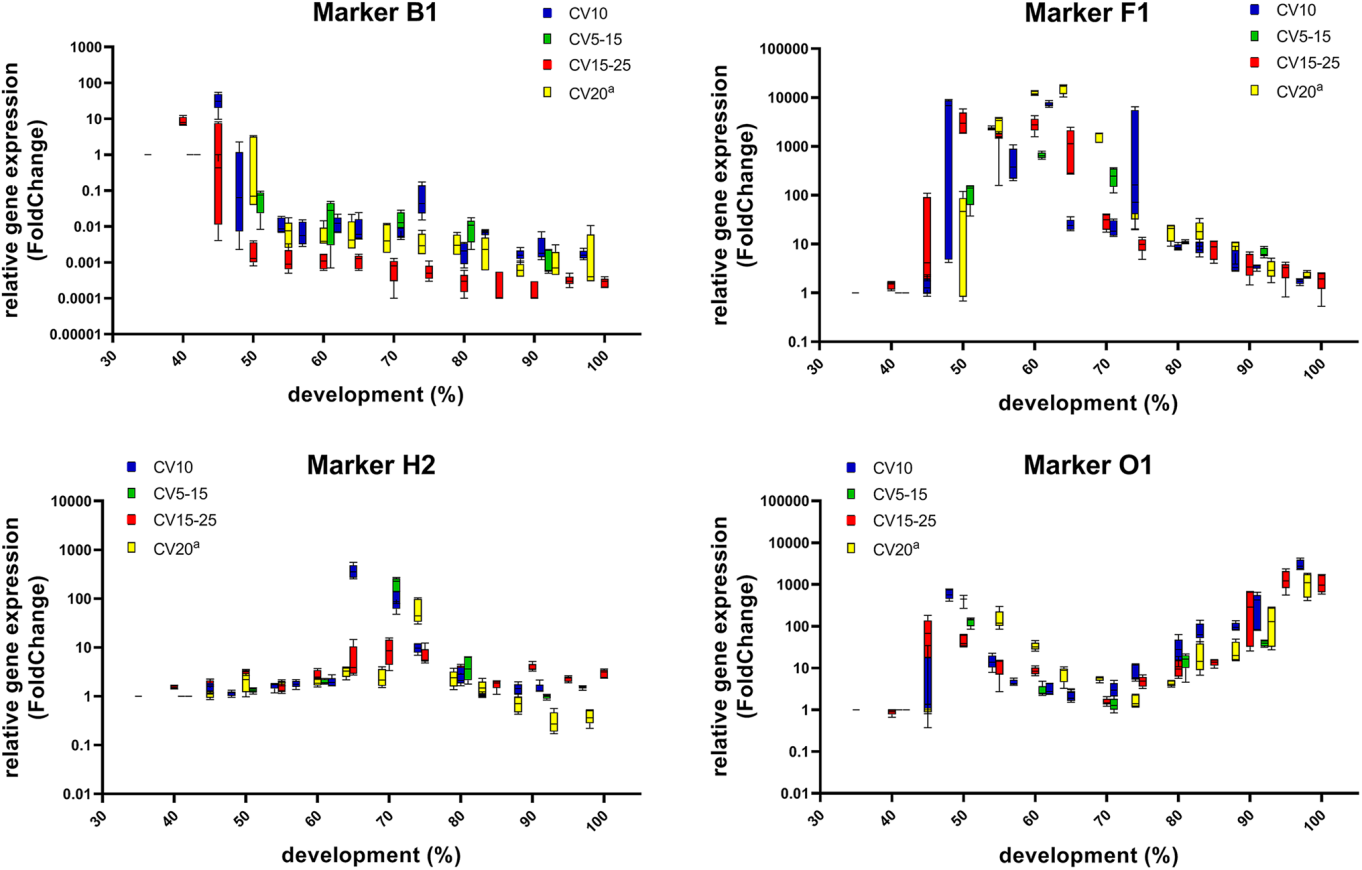
Gene expression data of the markers B1, F1, H2 and O1 of different *C.* *vicina* breeding (^a^data from [19]). The relative gene expression (fold change) during pupal development (%) are visualised in Tukey plots. One hundred percent development corresponds to the complete development from oviposition to eclosion of imago

In order to validate a method for age estimation of *C. vicina* pupae, the expression of the established marker genes in the *C.* *vicina* pupae bred at varying temperature conditions were analysed. First of all, it was examined whether the gene expression profiles of the pupae behave the same under constant or fluctuating conditions but at the same mean temperature. The gene expression patterns of four markers at each breeding temperature are shown as an example in Fig. 1. Figures for gene expression patterns of all markers at each breeding temperature, except CVO, which is only used to validate the age prediction tool, are shown in supplement (Supplementary Information, Figure S1).

As demonstrated in Table 1, the required ADD for immature development is temperature dependent. For a better comparison of gene expression under different temperature conditions, the development of the several breeding of *C.* *vicina* was therefore given as a total percentage development. The complete development, from oviposition to eclosion of imago, equates to 100%. This disregarded the condition-dependent differences in required ADD.

The fold change values, which represent the change in gene expression, ranged between approximately 0.0001 and 10,000. The expression patterns of the marker genes of *C. vicina* pupae bred at different temperature conditions (constant or fluctuating), but the same mean temperature (CV10 vs. CV5–15) is uniform during metamorphosis. Comparison of these gene expression data with the gene expressions of CV15–25 and the gene expressions of CV20 from Zajac et al. [19] reveals also similar expression patterns. Therefore, it could be confirmed that the expression patterns for each specific age marker are similar during pupal development at the different examined temperature profiles.

### Age prediction tool

To use the generated gene expression data to predict pupal age, an R-based tool was established. This tool consists of two scripts. The first script generates a database which is used in the second script to compare the gene expression data of a pupa with an unknown age. The mathematical strategy of the first script included the analysis of the gene expression data of each breeding separately and calculates the respective cubic spline interpolation of the expression of each marker. Thereby, every marker received its own specific function. After a leave-one-out cross-validation, the 50%, 75% and 95% confidence interval of the information content of these data for every pupal development age have been calculated.

**Fig. 2:**
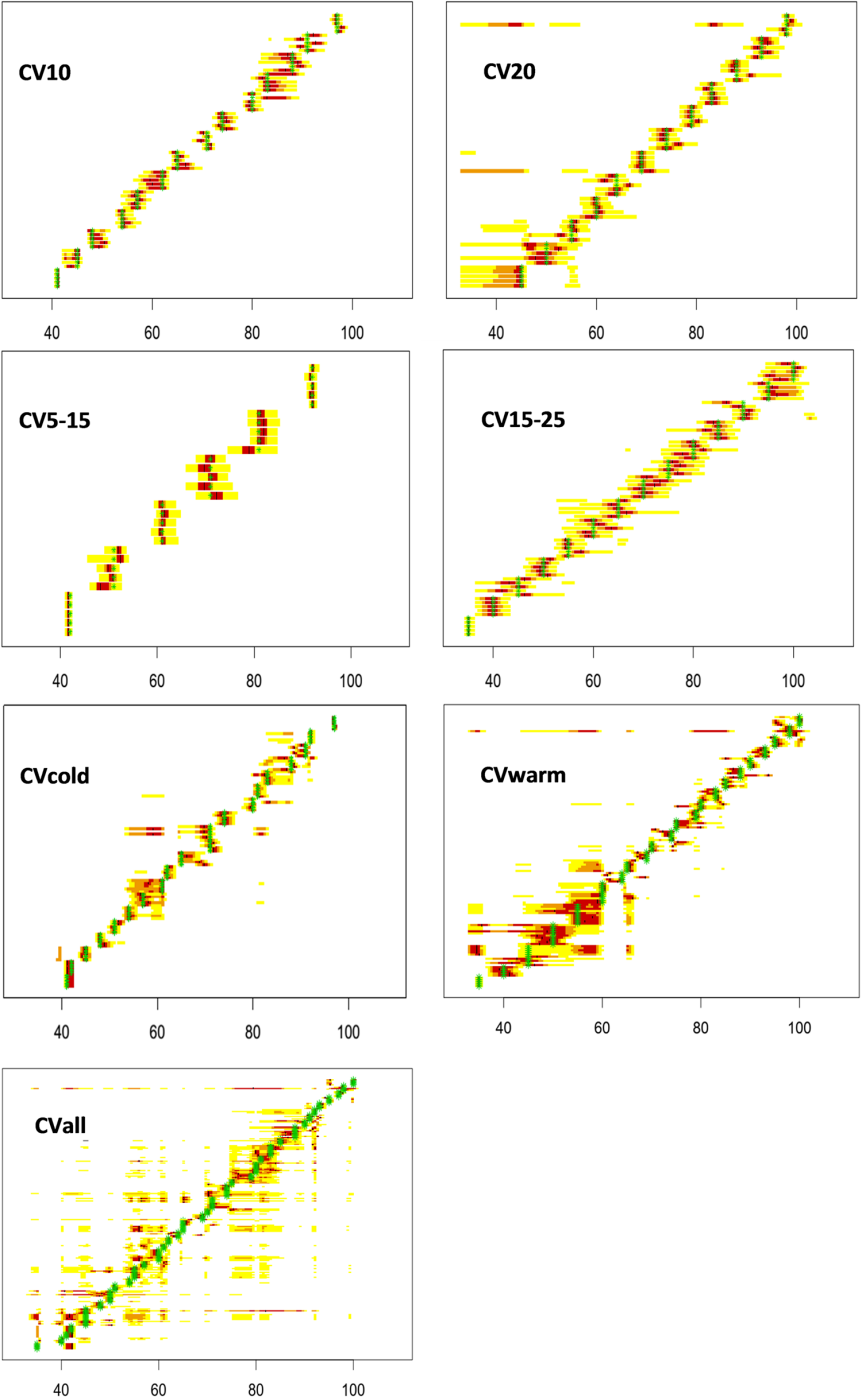
The 50%, 75% and 95% confidence intervals of each pupa of the different *C.* *vicina* breeding (CV10, CV20, CV5–15 and CV15–25) and of the pooled *C.* *vicina* breeding CV10 and CV5–15 (CVcold), CV20 and CV15–25 (CVwarm) and all breeding simultaneously (CVall). Each row represents the corresponding confidence intervals of one *C.* *vicina* pupa at a given development stage. Red areas represent the 50% confidence interval, orange areas represent the 75% confidence interval and yellow areas represent the 95% confidence intervals. The black lines indicate the expected value and green stars indicate the sampling time during pupal development

A total of 7 databases were generated to examine the gene expression: each breeding individually (CV10, CV5–15, CV20 and CV15–25), the pooled breeding of CV10 and CV5–15 (CVcold), the pooled breeding of CV20 and CV15–25 (CVwarm) and all four breeding simultaneously (CVall). In Fig. 2, the 50%, 75% and 95% confidence intervals of each pupa for these 7 databases are represented. For each development stage, the data of five pupae are given.

### Validation of the age prediction tool

**Fig. 3:**
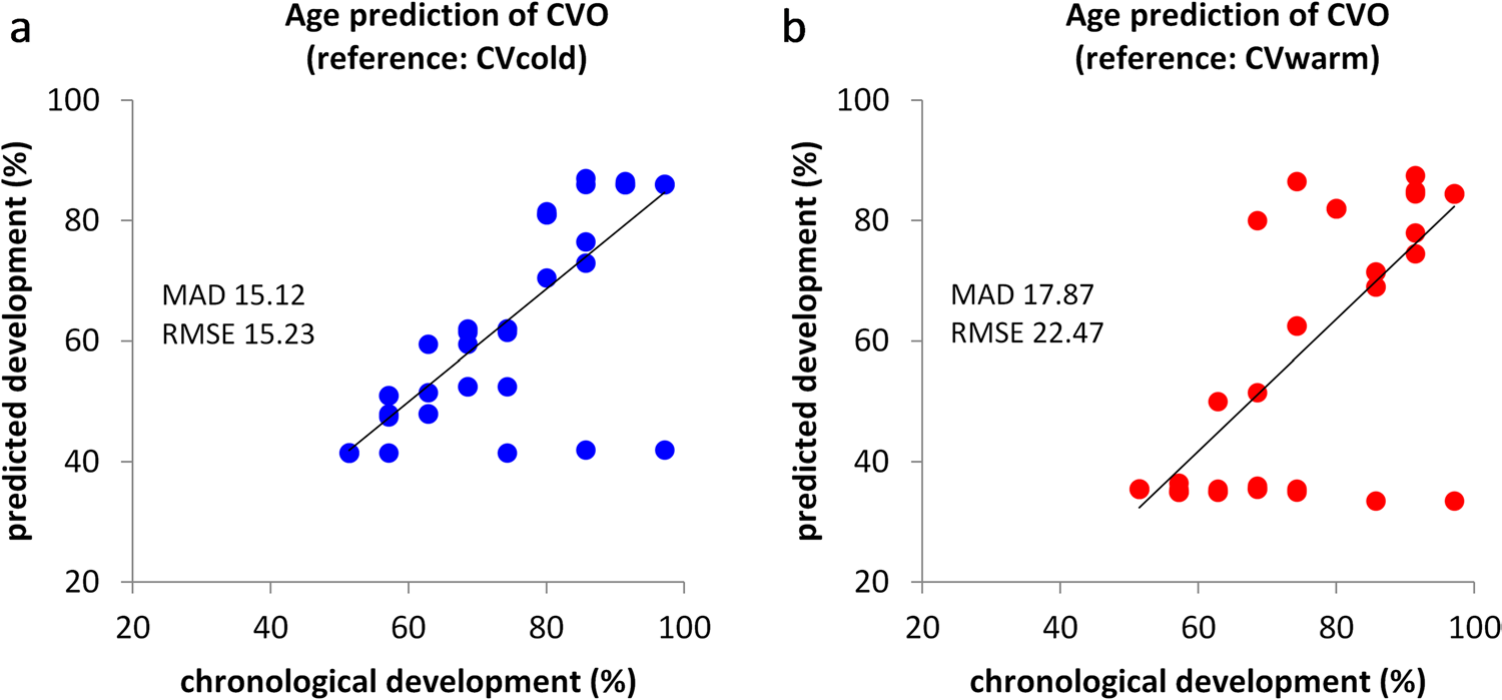
Age prediction of *C.* *vicina* pupae of the outdoor breeding (CVO) using the 28 molecular age markers. The development stage was estimated applying the age prediction tool with **a** CVcold and **b** CVwarm as reference data

For validation of the age prediction tool, the second script was used. The measured gene expression data of each *C. vicina* pupa of the outdoor breeding were imported into the second script of the age prediction tool and were compared to the analysed reference databases of script 1 (CVcold as well as CVwarm). The resulting confidence intervals cover the true age with a corresponding probability of 50%, 75% or 95%. For visualisation of the model performance, the mean values of the 95% CI of the predicted development were plotted against the mean of the actual development (Fig. 3). Age prediction of the outdoor breeding (CVO) using CVcold as reference dataset revealed a MAD (mean absolute deviation) of 15.12%-development and RMSE (root mean squared error) of 15.23%-development (Fig. 3a). Performing age prediction using CVwarm, a MAD of 17.87%-development and RMSE of 22.47%-development were resulted (Fig. 3b).

**Fig. 4:**
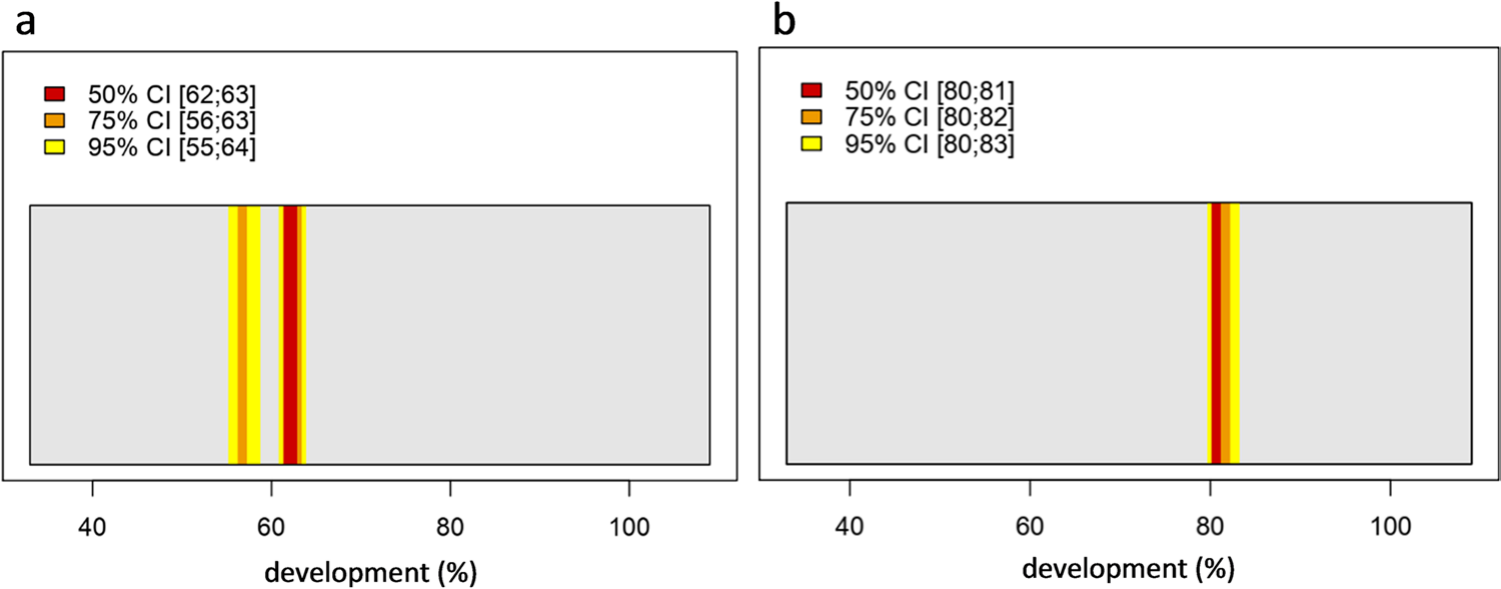
Application results of the age prediction tool of two *C.* *vicina* pupae (CVO) using CVcold as reference database. The output of script 2 demonstrates the estimated age in confidence interval bars. Red bars represent the 50%, orange bars the 75% and yellow bars the 95% confidence interval (CI). The legend lists the range of the respective CI (square brackets), which represents the estimated age in %-development. The true age of the exemplary pupae is **a** 60–66% and **b** 77–83%

As an example of script 2 output and calculation of PMI_min_, Fig. 4 presents plots of two pupae of CVO with a percentage development of 60–66% (Fig. 4a) and 77–83% (Fig. 4b). Since smaller and thus better MAD and RMSE values were obtained for CVO with the reference database CVcold instead of CVwarm, CVcold was used for the following exemplary calculation of PMI_min_. The estimated confidence intervals (CI) for the younger pupa are 62–63% (50% CI), 56–63% (75% CI) and 55–64% (95% CI), respectively. For the older pupa, the confidence intervals are 80–81% (50% CI), 80–82% (75% CI) and 80–83% (95% CI), respectively. Converting this development into ADD, we use the mean value of the 95% confidence interval and the mean value of total ADD for CV10 (567 ADD) and CV5–15 (487 ADD). This results in 314 ADD for the younger pupa and 430 ADD for the older pupa. Calculating the PMI_min_ from the predicted ADD, at an environmental temperature of 15 °C and a lower threshold of 2 °C, the result is 24 days for the younger pupa and 33 days for the older pupa.

## Discussion

Gene expression profiling is a useful tool for determination the age of *C. vicina* pupae, which allows conclusions of the PMI_min_. In the current study, the expression levels of age-specific genes of several bred *C. vicina* pupae were quantified to validate a RT-qPCR assay for age estimation. Continuing the study by Zajac et al. [19], the gene expressions of *C. vicina* pupae, who were bred at different temperature conditions (constant or fluctuating), were analysed and validated with the established molecular age markers.

### Development of *C. vicina*

In the present study the larvae died after eclosion at fluctuating temperatures of 25–35 °C. Reiter et al. [33] and Donovan et al. [34] also observed that larvae bred at constant temperature of 35 °C had died before reaching the pupal stage, which can be traced back to an unphysiological condition for living and development for *C. vicina* larvae. At constant temperature of 30 °C, the post-feeding larvae pupated, but all pupae died. In the study of Defilippo et al. [35], also no imagos hatched after pupation at 30 °C.

In contrast to the assumption of Greenberg and Kunich [27] that the relationship between rate of development and environmental temperature is linear within a certain temperature range, different thermal summations of the several breeding of *C. vicina* were observed in current study: we observed that the lower the ambient temperature, the higher the necessary amount of accumulated heat (ADD). Greenberg [8] also observed that the amount of ADD necessary for the development are significantly greater at unfavourable temperatures (e.g. 10 and 12.5 °C) but also described no statistically significant differences in the ADD at favourable temperatures, which begin at 19 °C for *C. vicina*, according to Greenberg.

The effect of temperature conditions (constant or fluctuating) on the development of different insects has been contradictorily observed. Ames and Turner [24] investigated the impact of low temperature episodes in the development of the *Calliphora* species *C. vicina* and *C.* *vomitoria* and observed a decreasing total ADD necessary for total development with increasing temperature. Moreover, for *Protophormia terraenovae* and *Lucilia sericata*, a faster development at fluctuating temperatures than at the same mean constant temperature was also described [20, 26]. In our study, we also observed a faster development of *C. vicina* under fluctuating temperature conditions. In contrast to our data, Davies and Ratcliffe [26] observed the need of a significant higher thermal summation for the larval development of *C.* *vicina* at fluctuating temperatures. Furthermore, a retarded development of *C. vicina* and *C. vomitoria* under fluctuating conditions was also described by Niederegger et al. [25]. Another study also showed a longer development time for *Aldrichina grahami* under fluctuating temperatures, in which a greater impact on *Aldrichina grahami* at low temperature periods than at high temperature periods was suspected [23]. Sert et al. [36] observed a longer total intrapuparial development of *Sarcophaga argyrostoma* under fluctuating temperature conditions compared to constant temperatures, but this retardation is not statistically significant. Colinet et al. [37] considered that the effect of fluctuating temperatures depends on the thermal mean and its closeness to the developmental thresholds. Furthermore, faster development under fluctuating temperature conditions seems to be the norm if the lower temperature is not harmful. The amplitude of the fluctuation also has an influence on the development time. For example, a great amplitude of fluctuation can reach the lower or upper temperature threshold, which has an effect on the development.

The percentage time for development of every breeding in the present study was between 35 and 43% from oviposition to pupation and between 57and 65% for pupal development. Similar development durations of *C. vicina* were also observed in previous studies [8, 32, 38]: 40–51% from oviposition to pupation and 49–60% from pupation to hatch. As a consequence, the percentage time of development allows a better comparability between different temperature conditions as in contrast to ADD, the development of *C.* *vicina* is given in %-development in the current study.

### Gene expression of *C. vicina* pupae

Age-dependent gene expression can be used to estimate the age of *C. vicina* pupae and, consequently, estimation of the PMI_min_. In this study, a qPCR-based gene expression profiling was performed to estimate the impact of temperature conditions (constant or fluctuating) on the age-dependent gene expression of *C.* *vicina* pupae. The molecular markers reflect the expression of age-dependent genes. Each marker is up- or downregulated at a particular time during pupal development and has an individual gene expression pattern regardless if temperature is constant or fluctuating. Zajac et al. [19] also described a temperature independency of these molecular age markers at three different constant temperatures (17 °C, 20 °C and 25 °C). Zajac et al. [18, 19] identified a large number of age-related markers (*n* = 30) of *C.* *vicina* for the first time. Other studies investigated the age-dependent gene expression in each case 4 markers for *Sarcophaga dux* [12], *Sarcophaga peregrina* [11] and *C. vicina* [15–17]. Liu et al. [14] analysed the gene expression of 3 markers for age estimation of *Aldrichina grahami* pupae. For age estimation of *Lucilia sericata*, 9 age-related markers were described [13]. By analysing more age markers of different expressed genes, age determination becomes more reliable and accurate.

The expression level of the molecular age markers during pupal development always refers to the gene expression on day 1 of pupation, whose fold change was set to value 1 in every breeding. Thus, fold change values above 1 represent increased gene expression, and values below 1 represent decreased gene expression of the molecular age markers compared to day 1 of pupation. The amount of gene expression alteration is different for each marker. The fold change values, ranging between approximately 0.0001 and 10,000, indicate high quantitative differences of gene expression. In contrast to previously described age markers, which only show fold change values of less than 15 [11–17], the analysed molecular age markers show a stronger change in gene expression during pupal development, which suggests a stronger predictive power.

### Age prediction tool

Based on the statistical data of the databases CV10, CV5–15 and CV15–25, an accurate and reliable age estimation of *C. vicina* pupae develop at this temperature conditions is possible. The CV20 database shows that the accuracy of age estimation decreases for pupae developing at 20 °C.

The databases of *C. vicina* pupae bred at different temperature conditions, but the same mean temperature (CVcold and CVwarm) shows broader confidence intervals, which indicate slightly less reliable age estimation of *C. vicina* pupae developed at these temperature conditions compared to the databases built on the data from each individual breeding. The higher spread for CVwarm may reflect slight differences in data collection of CV20 [19]. But all in all, CVcold and CVwarm show agreeable datasets for the age prediction tool. This confirms the assumption that age-dependent gene expression is equal at constant and fluctuating temperature conditions. The pooled database of all breeding (CVall) showed that no reliable age estimation of *C. vicina* pupae is possible using this database. Noting that percentage development was considered here, it suggests that there must be a temporal shift in gene expression during pupal development. This demonstrates that an accurate and reliable age estimation of *C. vicina* pupae with this age prediction tool is only practicable in defined temperature ranges (CVcold and CVwarm).

### Validation of the age prediction tool

For the aforementioned reason, only CVcold and CVwarm databases were used for validation. Therefor an outdoor breeding of *C. vicina* pupae (CVO) were analysed to constitute a more realistic scenario. The forecast quality of this outdoor collective resulted in a MAD of 15.12%-development and RMSE of 15.23%-development for CVcold as a database. For CVwarm as reference, the MAD was 17.87%-development and RMSE was 22.47%-development. In this case, CVcold as the reference database shows a higher accuracy of age prediction. However, both age prediction strategies show a positive correlation between chronological and predicted age, despite the deviating temperature conditions of CVO, whose temperature range was 8–27 °C with a mean temperature of 15 °C, from the reference databases (CVcold = 10 °C, CVwarm = 20 °C). The three values with a chronological development of approx. 74%, 86% and 97% and a prediction development of approx. 42% for CVcold and approx. 34% for CVwarm are conspicuous. Excluding these three *C. vicina* pupae from the validation of the age prediction model leads to a MAD of 14.63%-development and RMSE of 10.10%-development for CVcold and a MAD of 17.22%-development and RMSE of 18.41%-development for CVwarm. This illustrates once again that in order to ensure accurate and reliable age estimation of *C. vicina* pupae in casework, a reference database adjusted to the environmental temperature must be selected. Considering the mean month temperature of the DWD (Deutscher Wetterdienst) for 2020 in Frankfurt am Main, the following application is recommended: for outdoor corpses found in June–September (DWD: approx. 20 °C), using the CVwarm reference database, and for outdoor corpses found in October–May (DWD: approx. 8 °C), using CVcold.

Since the age prediction tool gives the age of a pupa in %-development, a conversion to ADD must be carried out for calculation of PMI_min_. However, in this study, it was observed that under different temperature conditions, *C. vicina* pupae did not require the same amount of ADD for complete development. Therefore, it is suggested that depending on the used reference database (implemented in script 1 of the age prediction tool), the respective mean amount of ADD should be used for conversion. For CVcold, this leads to a mean amount of 527 ADD for total development of *C. vicina*, for CVwarm 372 ADD.

The exemplary calculation of the PMI_min_ of the two *C. vicina* pupae in Fig. 4 resulted in 314 ADD for the younger and 430 ADD for the older pupa. The ADD actually required for these pupae were approx. 303 ADD and 395 ADD. These higher predicted ADD values are caused by the different mean temperatures: CVcold = 10 °C and CVO = 15 °C. The calculated PMI_min_ of 24 days for the younger pupa and 33 days for the older pupa is slight overestimated compared to the actual daily ages of the two pupae of 23 days and 29 days. This raises the question whether it is advisable to generate another database that covers the temperature range of 10–20 °C to avoid a systematic over- or underestimation for *C.* *vicina* pupae grew up at a mean temperature between 10 and 20 °C.

To determine the time since death in forensic casework, the species and age of an insect found on the corpse or in the surrounding environment is first identified. The physiological age is usually given in ADD or ADH. However, as described above, the ADD or ADH value is influenced by several factors, such as the thermal mean and the temperature condition. To ensure a reliable PMI_min_ calculation, the percentage development time should be used due to less variation between different environmental conditions, followed by a conversion to ADD. However, adequate total ADD (required ADD for total development) must be used depending on the ambient temperature range (total ADD for cold or warm temperatures).

## Supplementary Information

Below is the link to the electronic supplementary material.

Supplementary file1 (PDF 119 KB)

Supplementary file2 (PDF 15 KB)

Supplementary file3 (PDF 8 KB)

Supplementary file4 (PDF 18 KB)

## Data Availability

Not applicable.
