# Supplementary material for: Age-dependent gene expression of Calliphora vicina pupae (Diptera: Calliphoridae) at constant and fluctuating temperatures
Source: Int J Legal Med. 2021 Sep 27;135(6):2625–35. doi: 10.1007/s00414-021-02704-x (PMC8523437; doi:10.1007/s00414-021-02704-x)
Supplement: Supplementary file 1 — Supplementary file1 (PDF 119 KB) [file 414_2021_2704_MOESM1_ESM.pdf]

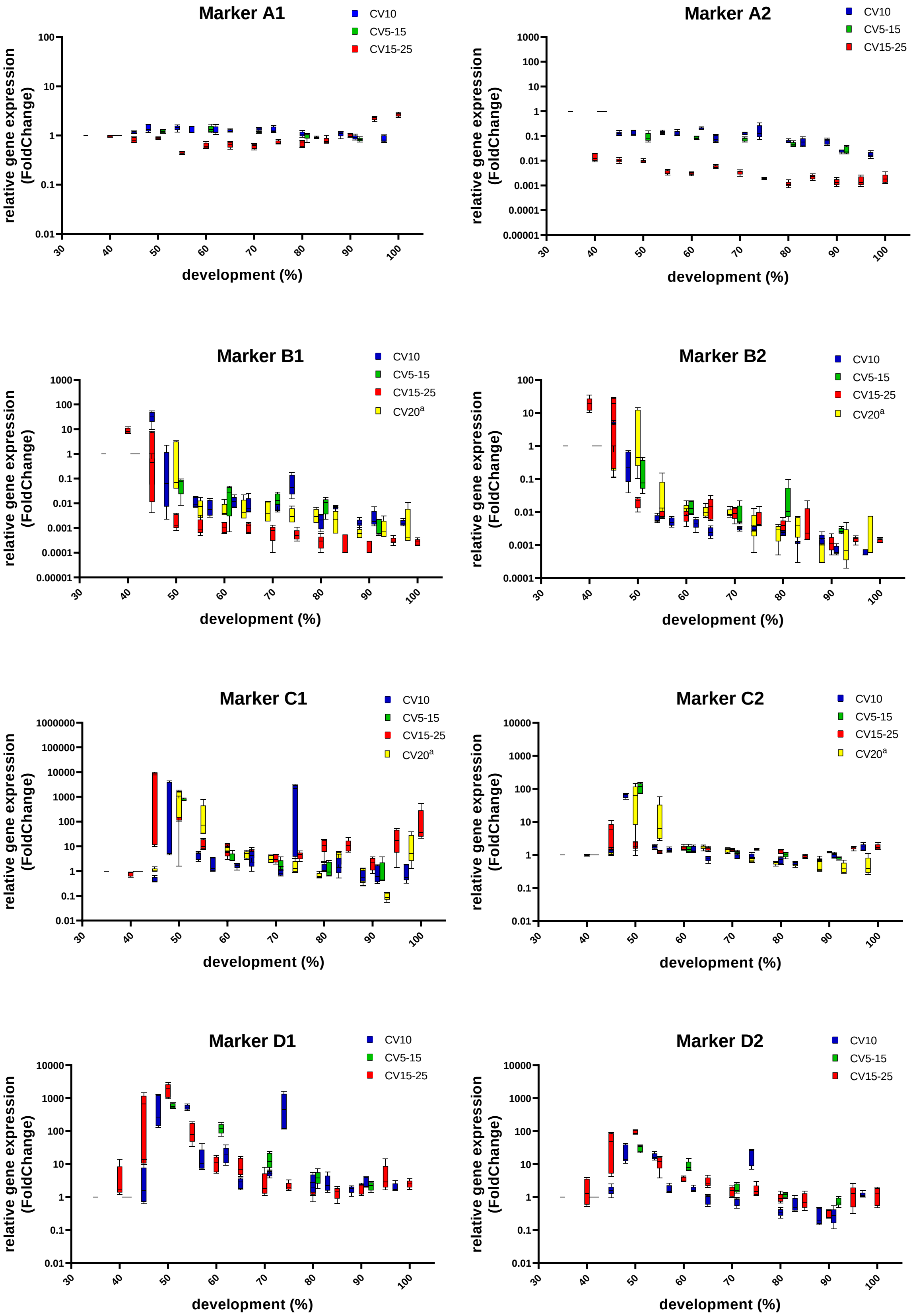

Marker A1
CV10
CV5-15
CV15-25
relative gene expression (FoldChange)
development (%)
Marker A2
CV10
CV5-15
CV15-25
relative gene expression (FoldChange)
development (%)
Marker B1
CV10
CV5-15
CV15-25
CV20a
relative gene expression (FoldChange)
development (%)
Marker B2
CV10
CV5-15
CV15-25
CV20a
relative gene expression (FoldChange)
development (%)
Marker C1
CV10
CV5-15
CV15-25
CV20a
relative gene expression (FoldChange)
development (%)
Marker C2
CV10
CV5-15
CV15-25
CV20a
relative gene expression (FoldChange)
development (%)
Marker D1
CV10
CV5-15
CV15-25
relative gene expression (FoldChange)
development (%)
Marker D2
CV10
CV5-15
CV15-25
relative gene expression (FoldChange)
development (%)

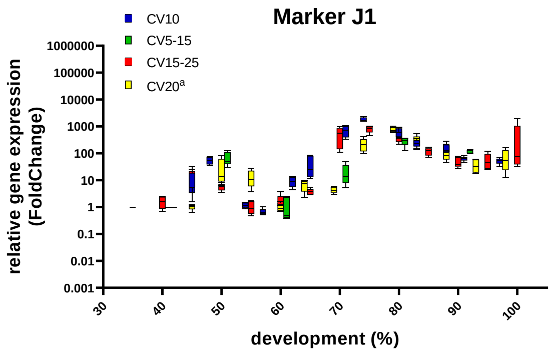
Marker J1
CV10
CV5-15
CV15-25
CV20a
relative gene expression (FoldChange)
1000000
100000
10000
1000
100
10
1
0.1
0.01
0.001
30
40
50
60
70
80
90
100
development (%)

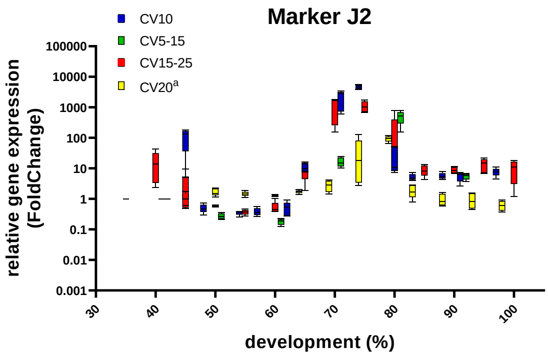
Marker J2
CV10
CV5-15
CV15-25
CV20a
relative gene expression (FoldChange)
100000
10000
1000
100
10
1
0.1
0.01
0.001
30
40
50
60
70
80
90
100
development (%)

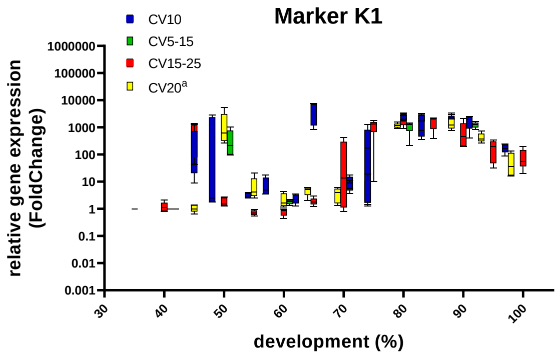
Marker K1
CV10
CV5-15
CV15-25
CV20a
relative gene expression (FoldChange)
1000000
100000
10000
1000
100
10
1
0.1
0.01
0.001
30
40
50
60
70
80
90
100
development (%)

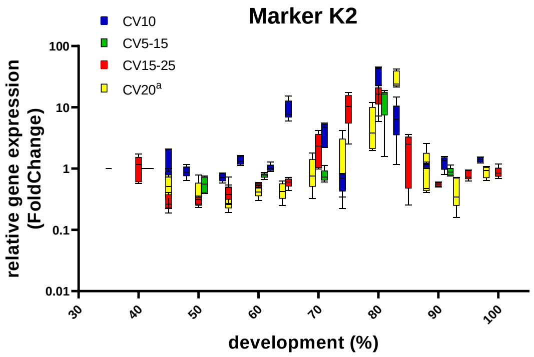
Marker K2
CV10
CV5-15
CV15-25
CV20a
relative gene expression (FoldChange)
100
10
1
0.1
0.01
30
40
50
60
70
80
90
100
development (%)

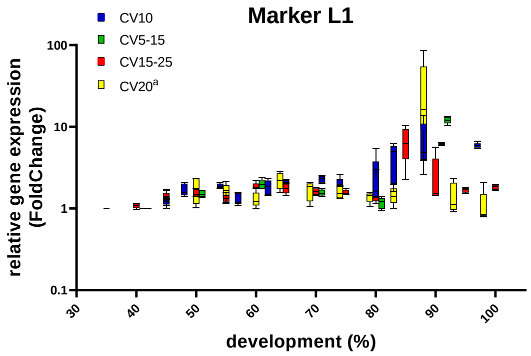
Marker L1
CV10
CV5-15
CV15-25
CV20a
relative gene expression (FoldChange)
100
10
1
0.1
30
40
50
60
70
80
90
100
development (%)

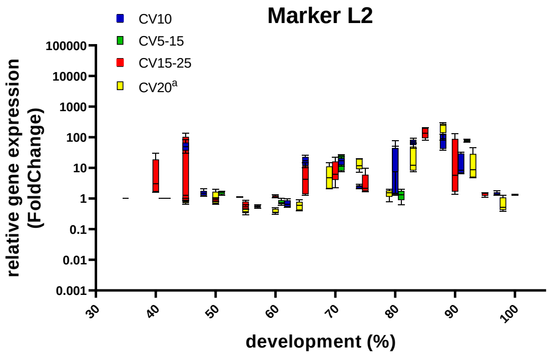
Marker L2
CV10
CV5-15
CV15-25
CV20a
relative gene expression (FoldChange)
100000
10000
1000
100
10
1
0.1
0.01
0.001
30
40
50
60
70
80
90
100
development (%)

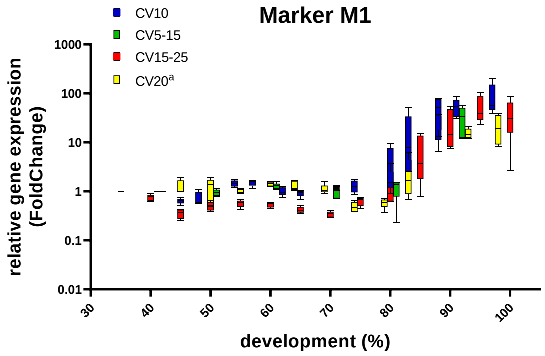
Marker M1
CV10
CV5-15
CV15-25
CV20a
relative gene expression (FoldChange)
1000
100
10
1
0.1
0.01
30
40
50
60
70
80
90
100
development (%)

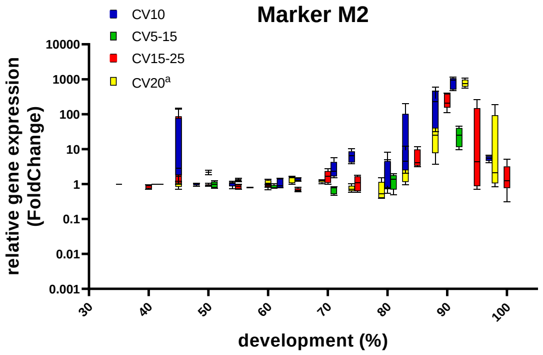
Marker M2
CV10
CV5-15
CV15-25
CV20a
relative gene expression (FoldChange)
10000
1000
100
10
1
0.1
0.01
0.001
30
40
50
60
70
80
90
100
development (%)

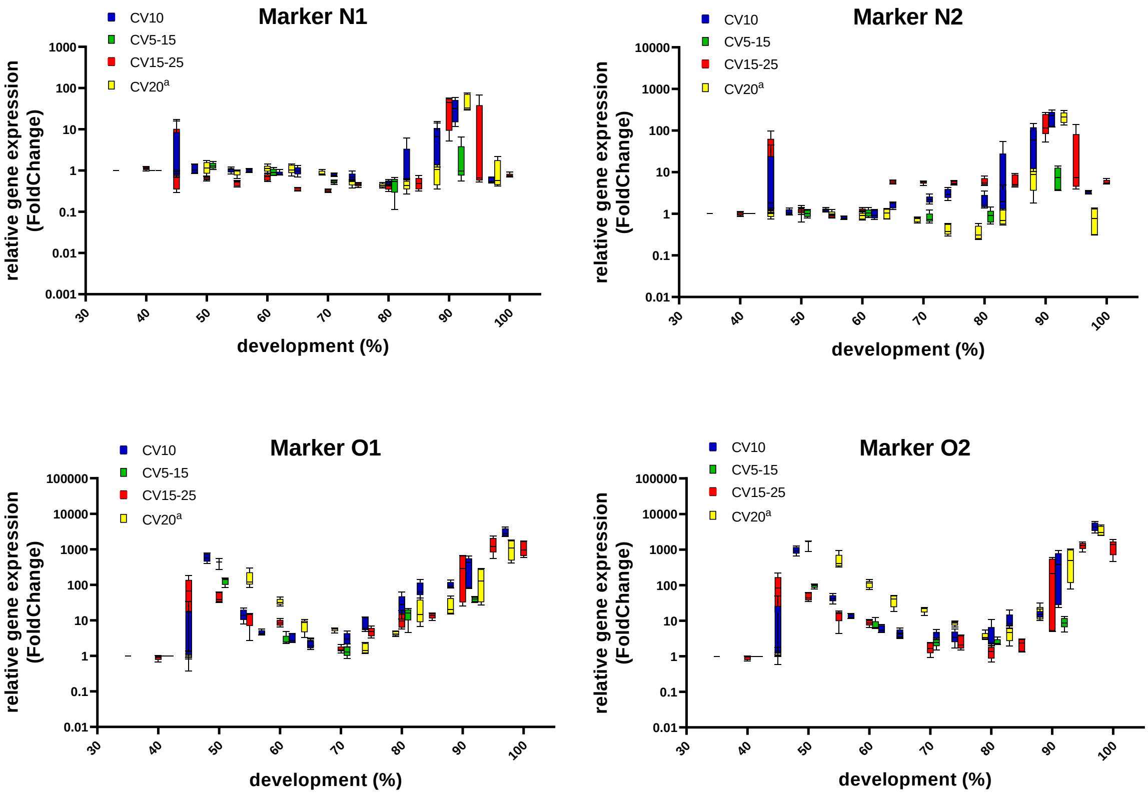

Supplementary Fig. 1 Gene expression data of each marker of different *C. vicina* breeding ([a] data from [19]). The relative gene expression (FoldChange) during pupal development (%) are visualized in Tukey plots. 100 % development corresponds to the complete development from oviposition to eclosion of imago
