# Supplementary material for: Age-dependent gene expression of Calliphora vicina pupae (Diptera: Calliphoridae) at constant and fluctuating temperatures
Source: Int J Legal Med. 2021 Sep 27;135(6):2625–35. doi: 10.1007/s00414-021-02704-x (PMC8523437; doi:10.1007/s00414-021-02704-x)
Supplement: Supplementary file 2 — Supplementary file2 (PDF 15 KB) [file 414_2021_2704_MOESM2_ESM.pdf]

Supplementary Table 1 Physiological age (ADD) and temperature (°C) of each day of the outdoor breeding (CVO).

ADD	Temperature (°C)
23	25.00
48	26.75
65	19.53
79	15.88
92	15.20
104	14.01
116	13.25
126	12.54
138	14.14
150	13.92
161	13.20
172	12.38
182	12.31
189	9.18
200	13.01
210	11.96
222	13.74
235	15.22
249	15.61
261	14.66
274	14.36
288	16.13
303	16.59
319	17.99
335	18.67
351	17.74
366	16.61
380	16.52
395	16.98
409	15.54
422	15.55
435	15.06
445	11.88
454	10.61
461	8.85

ADD, accumulated degree days
