# Supplementary material for: Age-dependent gene expression of Calliphora vicina pupae (Diptera: Calliphoridae) at constant and fluctuating temperatures
Source: Int J Legal Med. 2021 Sep 27;135(6):2625–35. doi: 10.1007/s00414-021-02704-x (PMC8523437; doi:10.1007/s00414-021-02704-x)
Supplement: Supplementary file 3 — Supplementary file3 (PDF 8 KB) [file 414_2021_2704_MOESM3_ESM.pdf]

Supplementary Table 2 New designed primer sequences used for marker D1 and D2 amplification in the RT-qPCR.

Marker	Forward primer	Reverse primer	Product Length (bp)	Contig	Annotation
**D1**	AATCGTGGGGATGTGGCAAA	TGTACCCACTGCTTTCACCG	146	contig3088	ref|XP_023306098.1| probable chitinase 10 [*Lucilia cuprina*]
**D2**	AGGCAGCAGTAGTGTCGAAT	CCGTCAAGCTCGTTTGGCTA	175	contig6281	ref|XP_023297597.1| seipin [*Lucilia cuprina*]
