# Supplementary material for: Age-dependent gene expression of Calliphora vicina pupae (Diptera: Calliphoridae) at constant and fluctuating temperatures
Source: Int J Legal Med. 2021 Sep 27;135(6):2625–35. doi: 10.1007/s00414-021-02704-x (PMC8523437; doi:10.1007/s00414-021-02704-x)
Supplement: Supplementary file 4 — Supplementary file4 (PDF 18 KB) [file 414_2021_2704_MOESM4_ESM.pdf]

Supplementary Table 3 Annotations of all age markers and reference gene using in this study. The markers were annotated using BLAST (Basic Local Alignment Search Tool).

Marker	Annotation
**A1**	No significant similarity found
**A2**	ref|XP_023295602.1| uncharacterized protein LOC111678465 [*Lucilia cuprina*]
**B1**	gb|KNC21978.1| hypothetical protein FF38_12141 [*Lucilia cuprina*]
**B2**	gb|KNC23606.1| hypothetical protein FF38_00967 [*Lucilia cuprina*]
**C1**	ref|XP_023309368.1| fibrous sheath CABYR-binding protein-like [*Lucilia cuprina*]
**C2**	ref|XP_023309368.1| fibrous sheath CABYR-binding protein-like [*Lucilia cuprina*]
**D1**	ref|XP_023306098.1| probable chitinase 10 [*Lucilia cuprina*]
**D2**	ref|XP_023297597.1| seipin [*Lucilia cuprina*]
**F1**	gb|KNC27587.1| hypothetical protein FF38_05100 [*Lucilia cuprina*]
**F2**	gb|TMW54454.1| hypothetical protein DOY81_000446 [*Sarcophaga bullata*]
**G1**	ref|XP_023305727.1| uncharacterized protein LOC111687521 [*Lucilia cuprina*]
**G2**	gb|KNC30751.1| hypothetical protein FF38_11721 [*Lucilia cuprina*]
**H1**	gb|KNC29875.1| hypothetical protein FF38_13502 [*Lucilia cuprina*]
**H2**	gb|KNC24893.1| hypothetical protein FF38_03014 [*Lucilia cuprina*]
**I1**	ref|XP_023308080.1| splicing factor, arginine/serine-rich 19-like [*Lucilia cuprina*]
**I2**	gb|KNC32097.1| hypothetical protein FF38_00012 [*Lucilia cuprina*]
**J1**	ref|XP_023298844.1| neuropeptide-like 3 [*Lucilia cuprina*]
**J2**	ref|XP_023304353.1| histidine-rich glycoprotein-like [*Lucilia cuprina*]
**K1**	ref|XP_023306348.1| pupal cuticle protein Edg-78E-like [*Lucilia cuprina*]
**K2**	ref|XP_023296676.1| beta-glucuronidase isoform X2 [*Lucilia cuprina*]
**L1**	gb|KNC32423.1| hypothetical protein FF38_04989 [*Lucilia cuprina*]
**L2**	ref|XP_023309487.1| hornerin [*Lucilia cuprina*]
**M1**	ref|XP_023301633.1| lectizyme-like [*Lucilia cuprina*]
**M2**	ref|XP_023295851.1| cuticle protein 18.6 [*Lucilia cuprina*]
**N1**	gb|KNC31140.1| hypothetical protein FF38_13965 [*Lucilia cuprina*]
**N2**	ref|XP_023295851.1| cuticle protein 18.6 [*Lucilia cuprina*]

**O1**	No significant similarity found
**O2**	ref|XP_023300406.1| vitelline membrane protein Vm26Ab-like [*Lucilia cuprina*]
**R2**	ref|XP_023309093.1| electron transfer flavoprotein subunit beta [*Lucilia cuprina*]
